# Cardiac Output Measurements in Septic Patients: Comparing the Accuracy of USCOM to PiCCO

**DOI:** 10.1155/2012/270631

**Published:** 2011-11-29

**Authors:** Sophia Horster, Hans-Joachim Stemmler, Nina Strecker, Florian Brettner, Andreas Hausmann, Jitske Cnossen, Klaus G. Parhofer, Thomas Nickel, Sandra Geiger

**Affiliations:** ^1^Medical Department II, Ludwig Maximilian University of Munich, Campus Großhadern, Marchioninistraße 15, 81377 Munich, Germany; ^2^Medical Department III, Ludwig Maximilian University of Munich, Campus Großhadern, Marchioninistraße 15, 81377 Munich, Germany; ^3^Department of Anesthesia II, Ludwig Maximilian University of Munich, Campus Großhadern, Marchioninistraße 15, 81377 Munich, Germany; ^4^Medical Department I, Ludwig Maximilian University of Munich, Campus Großhadern, Marchioninistraße 15, 81377 Munich, Germany

## Abstract

USCOM is an ultrasound-based method which has been accepted for noninvasive hemodynamic monitoring in various clinical conditions (USCOM, Ultrasonic cardiac output monitoring). The present study aimed at comparing the accuracy of the USCOM device with that of the thermodilution technique in patients with septicemia. We conducted a prospective observational study in a medical but noncardiological ICU of a university hospital. Septic adult patients (median age 55 years, median SAPS-II-Score 43 points) on mechanical ventilation and catecholamine support were monitored with USCOM and PiCCO (*n* = 70). Seventy paired left-sided CO measurements (transaortic access = CO_US-A_) were obtained. The mean CO_US-A_ were 6.55 l/min (±2.19) versus CO_PiCCO_ 6.5 l/min (±2.18). The correlation coefficient was *r* = 0.89. Comparison by Bland-Altman analysis revealed a bias of −0.36 l/min (±0.99 l/min) leading to a mean percentage error of 29%. USCOM is a feasible and rapid method to evaluate CO in septic patients. USCOM does reliably represent CO values as compared to the reference technique based on thermodilution (PiCCO). It seems to be appropriate in situations where CO measurements are most pertinent to patient management.

## 1. Introduction

Thermodilution cardiac output measurements have been routinely performed as part of intensive care practice since the introduction of the balloon-directed, thermistor-tipped pulmonary artery catheter in the 1970s [1–3]. Introduced by Swan and Ganz, the pulmonary artery catheter (PAC) became to be the gold standard for more than two decades [1, 2]. However, arrhythmia, infection, and possible pulmonary artery disruption have always been concerns related to the use of a PAC and led to a growing interest in the development of noninvasive hemodynamic monitoring devices [4–6]. One less invasive thermodilution-based technique consists of the pulse-induced cardiac output device (PiCCO) but exclusively ultrasound-based devices as the USCOM monitor are entirely non-invasive methods for measuring CO [7–13]. Beside accuracy and the method-related risks, another crucial criterion consists of the time required for the determination of CO [14]. USCOM is a feasible, continuous-wave Doppler-based method which noninvasively measures CO in a fast and economical way.

The present study aimed at comparing the accuracy of the USCOM device with that of the thermodilution technique (PiCCO) in septic patients.

## 2. Materials and Methods

Seventy adult, predominantly and mechanically ventilated, patients were investigated in this observational study. All patients suffered from septic infections and required catecholamine support. The study protocol was approved by the institutional ethics committee. As the protocol was the considered part of the routine practice, informed consent was waived.

All patients were measured by PiCCO and USCOM (CO_US-A_ left-sided aortal access *n* = 70). With the assistance of a nurse, CO measurements (CO_USCOM_, CO_PiCCO_) were carried out simultaneously. All measurements were undertaken during patients were hemodynamically stable throughout the time of CO measurements. The PiCCO device was recalibrated immediately prior to any measurements by USCOM. To exclude an interindividual observer variability, all CO measurements by USCOM and PiCCO were undertaken by the same investigator.

### 2.1. USCOM

The USCOM device (USCOM Ltd., Sydney, Australia) is a non-invasive bedside method to evaluate cardiac output basing on continuous-wave Doppler ultrasound. After starting the USCOM device, the left-sided transaortic (CO_USA_) or right-sided transpulmonary access has to be chosen before the patients data like height, weight, and gender are typed in. The flow profile is obtained by commonly using a 2.2 MHz transducer placed on the chest in either the left parasternal position to measure transpulmonary blood flow (right-sided access, 3rd to 5th parasternal intercostal space) or the suprasternal position to measure transaortic blood flow (left-sided access, suprasternal notch). The operator registries a Doppler flow curve with maximal blood flow which is characterized by a sharp, well-defined waveform with the clearest audible sound. The flow profile is displayed as a time velocity curve at the monitor (VTI: velocity time integral). Once the optimal flow profile is obtained, the trace is frozen. The USCOM device calculates CO by the product of stroke volume (SV) and heart rate (HR) where the SV is the product of the velocity time integral (VTI) and the cross-sectional area of the chosen valve (CSA). The chosen valve cross-sectional area is given by the USCOM internal algorithm based on the formerly typed in patients data (height and gender) [15, 16].

### 2.2. PiCCO

Continuous cardiac output using pulse contour analysis was measured by the PiCCO plus system (Pulsion Medical Systems, Munich, Germany). Cardiac output was measured discontinuously by thermodilution using a triplicate injection of 15 mL ice-cold 0.9% saline administered through a temperature detecting inline sensor central vein catheter [17]. A femoral or brachial artery catheter (4-F aortic catheter with an integrated thermistor) registers the time until the bolus attains and identifies the alteration of temperature [18].

### 2.3. Statistical Analysis

The Bland-Altman Plot was used to estimate the bias and limits of agreement between measurements by the two methods [19]. According to the recommendations by L. A. H. Critchley and J.  A.  H. Critchley, we quoted the mean CO (*μ*), the bias, the limits of agreement (95% CI), and the percentage error (±2 SD/*μ*) [20]. Bland-Altman plots and correlation curves were performed using GraphPad for Windows (Version 5.01, GraphPad Software, San Diego, California, USA).

For statistical calculations (Pearsons' correlation coefficient) SPSS for Windows was used (Version 15.0, SPSS Institute, Chicago, Ill, USA).

## 3. Results

### 3.1. Patient Characteristics

Seventy mechanically ventilated patients with a catecholamine support (median norepinephrine 0.55 mg/h c.i., range 0.1–3.0) at a median age of 45 years and a median SAPS Score of 43 points were enrolled. The majorities of the patients suffered from hematological (*n* = 38) or hepatological diseases (*n* = 16). In 9 cases, patients had received prior chemotherapy- for solid tumors (*n* = 9), and 7 patients suffered from other diseases. All patients fulfilled the criteria for sepsis. In most cases sepsis was related to chemotherapy-induced neutropenia. Detailed patients' characteristics are given in Table 1.

### 3.2. Detection Ability: USCOM

In total, 70 left-sided, transaortic CO measures from 70 subjects were acquired. High-quality, left-sided transaortic doppler signals could not be obtained in two patients due to anatomic variability (short neck and tracheostoma). The detection ability rate was CO_US-A_ 98.4%.

### 3.3. USCOM versus PiCCO

#### 3.3.1. Transaortic Analysis: CO_US-A_

The CO values of seventy patients were measured by PiCCO and left-sided transaortic USCOM (126 paired measurements). 

**Figure 1:**
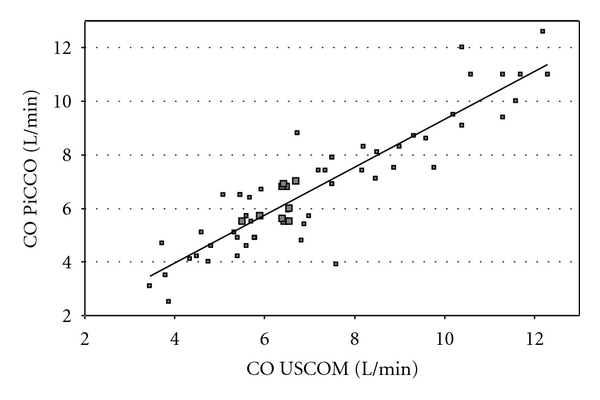
Correlation of CO measurements by USCOM and PiCCO (median CO USCOM 6.55 L/min ±2.19, median CO PiCCO 6.5 L/min ±2.18; *r* = 0.89) *(increased size of points which are multiples). *

The median CO was 6.5 l/min (±2.18) for PiCCO device and 6.55 l/min (±2.19) for the transaortic measurements with USCOM. The Pearsons' correlation coefficient was *r* = 0.89 (*P* < 0.01) (Figure 1).

**Figure 2:**
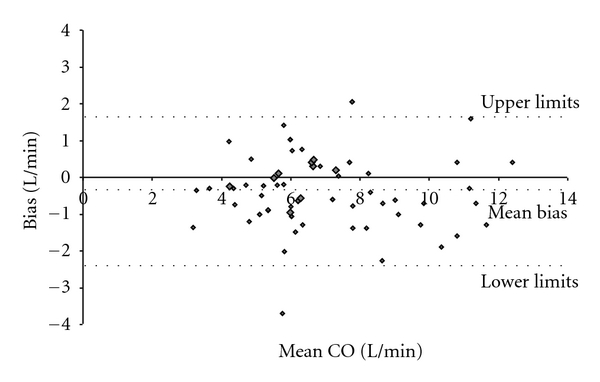
Bland-Altmann plot of left-sided, aortal CO measurements by USCOM versus PiCCO. The mean bias was −0.36 l/min ± 0.99 with 95% limits of agreement from −2.34 to 1.62. The percentage error according to Critchley and Critchley was 29%.

The bias, using the Bland-Altman analysis, was −0.36 l/min (±0.99 l/min) with 95% limits of agreement from −2.34 to 1.62 (Figure 2). The mean percentage error according to Critchley L. A. H. and J. A. H. Critchley amounts to 29%.

#### 3.3.2. Time Requirement: t_CO US-A_ versus t_CO PiCCO_

The time requirements for each single method of CO measurements were recorded (starting the device: first admissible result) on the following preconditions: 

PiCCO artery and central venous line were already in situ. The PiCCO device was recalibrated immediately prior to measurements. 

Mean measurement time of PiCCO-(t_CO PiCCO_) was 8.46 minutes (min) (±2.15; min/max 4.0/20.0 min) and of transaortic USCOM (t_CO US-A_) analysis 3.69 min (±1.59; min/max 1.0/10.0 min).

## 4. Discussion

This study aimed to compare the accuracy of CO measurements between the noninvasive continuous-wave Doppler-based monitoring system USCOM and a thermodilution-based technique (PiCCO).

USCOM is a noninvasive cardiac output monitor based on the transthoracic measurement of Doppler flow velocity over the aortic and pulmonary outflow tract. It is easy to operate, and CO is displayed “beat by beat”. Following a short booting time, the device can be used immediately. Moreover, the technique is reported to be easily learned after a short learning period by nonphysicians [21, 22].

In contrast to previously reported trials which investigated USCOM in predominantly cardiac surgical patients' collectives, we analyzed patients with sepsis. A former pilot study indicated a comparable accuracy of USCOM and the PiCCO device in a similar patients subset [23]. According to these data, the present study indicated also an acceptable agreement between the USCOM CO measurements and those determined by a thermodilution-based method.

For analyzing the accuracy, the Bland-Altman method was used because it measures the extent of deviation from the line of complete agreement (no bias) between the methods. This is different from the correlation coefficient which measures how close to a straight line the pairs of measurements lie, but that line need not to be the one of complete agreement. Moreover, in addition to reporting the mean cardiac output (*μ*), the bias, and the limits of agreement (95%CI), we quoted the percentage error as recommended by, L. A. H. Critchley and J. A. H. Critchley [20].

Analysing the accuracy of CO_US_ and CO_PiCCO_, the Pearsons' correlation coefficient was 0.89 which seems comparable to that reported in the study of Knobloch and coworkers. They investigated 36 patients by PAC and USCOM and obtained a comparable correlation coefficient of *r* = 0.87 (*P* < 0.01) [12]. By analyzing our data with the Bland-Altman method, the mean percentage error according to L. A. H. Critchley and J. A. H. Critchley was 29% for the transaortic access. Since the accepted threshold is <30% one can conclude that transaortic CO measurements by USCOM do reliably reflect the measurements by PiCCO.

In contrast to these data, an inferior accuracy for USCOM was reported by other authors who found that CO measurements by USCOM do not reliably represent absolute values as compared to pulmonary artery catheter thermodilution technique [16, 24]. Possible explanations for such incoherent findings are as follows.

1. Parts of reported examinations were done during cardiac surgery by placing the probe directly on the right ventricular outflow tract. Patients in our study, for instance, were ventilated mechanically which contributes to difficulties in CO measurements by an ultrasound-based device.
2. In cases of relatively high cardiac output, USCOM tends to underestimate the real CO value when it is relatively high [9–11]. On the contrary, such a difference does not appear in Su et al.'s research [10, 11]. They investigated patients with liver cirrhosis because of their unique hyperdynamic status with high CO values ranging up to 13.6 L/min. They found that even at high CO values, USCOM still reliably measures CO [10, 11].
3. The accuracy of the USCOM depends on obtaining accurate VTI and valve diameter measurements. An accurate VTI measurement requires a good flow signal. An inadequate beam alignment with the blood flow direction will lead to suboptimal Doppler signal.
4. The cross-sectional area of the chosen valve contributes to the estimated CO (CO = HR × SV; SV = VTI × CSA). The valve area is given by the height-based algorithm built into the device. Knirsch et al. studied twenty-four pediatric patients with congenital heart disease without shunt undergoing cardiac catheterization under general anesthesia [16]. Interpreting the moderate accuracy of USCOM in their study, it has to be considered that the USCOM algorithm which determinates the valve cross-sectional area based on the data of healthy volunteers [15]. Despite the opportunity to correct the valve cross-sectional area manually in cases of known cardiac valve anomalies after exact evaluation by transthoracic or transesophageal echocardiography, the first examination by USCOM can be misleadingly too low or too high.
5. The PiCCO device may be not as accurate as reference technique in this setting (septicemic patients). Any bias and limits of agreement observed in this study could therefore be explained by the inaccuracy of the PiCCO system. The accepted clinical standard is still the intermittent thermodilution technique which in has its own inherent variability [25–27].

Early goal-directed therapy (EGDT) has become regarded as the standard of care for the management of patients with severe sepsis and septic shock [14, 28, 29]. However, it is critical to discuss that the concept of EGDT is still an issue of controversy [30]. Nevertheless, USCOM is attractive in many ways. It is easy to use, and as an ultrasound technique is safe so it can be used repeatedly to measure the trend over time. It avoids the problems of an esophageal probe and is tolerated by awaken patients. Moreover, by using the USCOM device the physician will obtain a result in an unbeatable period of time. The role of USCOM is evolving but USCOM is limited to CO measurements and does not provide variables as pressure measurements or ScvO_2_. Thus, USCOM does not replace invasive methods as PiCCO or PAC. But USCOM seems to be appropriate in situations where CO measurement is most pertinent to patient management.

##  Author's Contribution

Sophia Horster and Sandra Geiger contributed equally to this work. 

## Figures and Tables

**Table 1:** Patient characteristics.

*n* = 70	Value/median	range	Standard deviation (+/−SD)
*Baseline characteristics*			
Age	45 years	23–78	
Gender	45 m/25 f		

*ICU characteristics*			
SAPS II score	43	23–60	7.14
BP (systolic)	124 mmHg	94–170	19.78
BP (diastolic)	58 mmHg	37–70	21.62
HR	97 bpm	53–142	20.0
CVP	10 mbar	3–17	5.02
Norepinephrine	0.5 mg/h	0.1–3.0	2.01
Mechanically ventilated	70		
fiO_2_	0.5	0.3–1.0	

*Hepatological disease*	*16*		
Liver cirrhosis	12		
SBP	5		
Hepatitis	1		
GI bleeding	3		
Pneumonia	2		
HCC	1		
Acute liver failure	4		
Liver transplantation	2		

*Haematological disease*	*38*		
Acute leukaemia	12		
SCT	6		
Chronic leukaemia	4		
Lymphoma	11		
Myeloma	5		

*Solid tumors*	*9*		
GI cancer	5		
Breast cancer	3		
Lung cancer	1		

*Other*	*7*		

*Abbreviations:* BP: blood pressure, HR: heart rate, CVP: central vein pressure, SBP: spontaneous bacterial peritonitis, HCC: hepatocellular carcinoma, SCT: stem cell transplantation, and GI: gastrointestinal.
